# Analytical studies on ascosin, candicidin and levorin multicomponent antifungal antibiotic complexes. The stereostructure of ascosin A2

**DOI:** 10.1038/srep40158

**Published:** 2017-01-09

**Authors:** Paweł Szczeblewski, Tomasz Laskowski, Bartosz Kubacki, Marta Dziergowska, Magda Liczmańska, Jakub Grynda, Paweł Kubica, Agata Kot-Wasik, Edward Borowski

**Affiliations:** 1 Department of Pharmaceutical Technology and Biochemistry, Faculty of Chemistry, Gdańsk University of Technology, Gabriela Narutowicza Str. 11/12, 80-233 Gdańsk, Poland; 2 Department of Analytical Chemistry, Faculty of Chemistry, Gdańsk University of Technology, Gabriela Narutowicza Str. 11/12, 80-233 Gdańsk, Poland

## Abstract

In the class of polyene macrolides, there is a subgroup of aromatic heptaenes, which exhibit the highest antifungal activity within this type of antibiotics. Yet, due to their complex nature, aromatic heptaenes were not extensively studied and their potential as drugs is currently underexploited. Moreover, there are many inconsistencies in the literature regarding the composition and the structures of the individual components of the aromatic heptaene complexes. Inspired by one of such cases, herein we conducted the analytical studies on ascosin, candicidin and levorin using HPLC-DAD-(ESI)Q-TOF techniques. The resulting chromatograms and the molecular masses of the individual components of these three complexes strongly indicated that the major components of ascosin, candicidin and levorin are structurally identical. In order to validate these results, the main component of previously structurally uncharacterized ascosin was derivatized, isolated and subjected to 2D NMR studies. The resulting structure of the ascosin’s main component, herein named ascosin A2, was shown to be identical with the earlier reported structures of the main components of candicidin and levorin complexes: candicidin D and levorin A2. In the end, all the structural knowledge regarding these three antibiotic complexes was gathered, systematized and completed, and the new nomenclature was proposed.

Polyene macrolides, membrane active antifungal antibiotics^1^,^2^, are leading and most prospective antifungal agents used for the treatment of systemic and topical fungal infections. This is due to their unique, among antifungal drugs, properties comprising: high antifungal activity, broad antifungal spectrum, fungicidal action, inability to induce resistant strains and overcoming the multidrug resistance (MDR) of fungi^3^. Compounds referred to as ‘polyene macrolides’ consist of a macrolide (macrocyclic lactone) ring containing a polyene chromophore and a polyol chain, and at least one glycosidically bound monosaccharide moiety. Depending on the size of the chromophore, polyene macrolides are classified as trienes, tetraenes, pentaenes, hexaenes, heptaenes and octaenes.

The most interesting and prospective group among the polyene macrolides are the heptaenes due to their clinical use potential. So far, the most successful compound of this group, Amphotericin B, after over 60 years of its use, is still considered to be “the lifesaving drug” and is called “the golden standard” for antifungal agents. Also, there is an interesting subclass of heptaene macrolides, which is called the aromatic heptaenes^1^,^2^,^4^. Members of this group are structurally similar to the Amphotericin B. The major structural difference is the presence of an aromatic sidechain attached to the macrolactone ring. Moreover, aromatic heptaenes contain heptaene chromophores of different geometries in comparison to the one of Amphotericin B (*cis-trans* geometry in aromatic heptaenes versus *all-trans* geometry in non-aromatic heptaenes)^5^.

The aromatic heptaene group of polyene macrolides also bear the great potential for the drug design. The strong encouragement to examine and develop these compounds is the highest of all polyene macrolides antifungal activity, which is over one order of magnitude higher than the activity of “the golden standard” Amphotericin B^6^,^7^. However, this potential has not been yet fully exploited due to the essential technical drawback. These antibiotics are produced by *Streptomycetes* as multicomponent complexes of closely related compounds, very difficult for isolation in the preparative scale. Consequently, the individual components are not available commercially. Moreover, there are many dissonances in the literature regarding the composition of the aromatic heptaene complexes, as well as most of the structures of their individual components are still unknown.

In the field of antibiotics, the rediscovery of previously reported agents occurs quite often, causing certain inconsistencies in the literature. The aim of this article is to solve one of such cases, regarding the issue of the similarity of the three aromatic heptaene complexes, namely ascosin, candicidin and levorin, which was raised repeatedly in the previous studies^8^,^9^,^10^,^11^,^12^.

Candicidin, produced by *Streptomyces griseus IMRU 3570*, was the first aromatic heptaene antibiotic complex to be discovered. It is also the most widely known complex in this class of polyene macrolides^13^,^14^. In the second half of XX century, it was temporarily used in medicine in the treatment of vaginal fungal infections. According to the literature, candicidin complex consist of at least three^15^, four to five^10^ or nine components, designated as candicidin A to I^16^, of which only the main component, candicidin D (Fig. 1), was structurally characterized (gross structure in 1979, stereostructural studies in 2015)^17^,^18^.

Levorin^19^,^20^, produced by *Actinomyces levoris (Krass)*, is used in the topical treatment of vulvovaginal candidiasis. This complex consists of five antibiotics, designated as levorin A0 to A4^8^,^21^, of which the gross structure of the main component, namely levorin A2, was shown to be identical with the gross structure of candicidin D^17^. The structures of levorin A1 and A3 with partially defined stereochemistry are also available (Fig. 2)^22^,^23^. In the literature, the structure of levorin A0 and another version of the structure of levorin A1 were also reported and cited repeatedly, yet they are considered to be erroneous due to the misinterpretation of the MS data.

Finally, ascosin, produced by *Streptomyces canescus*, was studied the least of these three complexes. Discovered in 1952, for the following 30 years was considered to be a homogenous substance^24^,^25^. In the 1980s the complex nature of ascosin was revealed, yet due to the very limited amount of studies neither the number of the individual components nor the nomenclature were defined^10^. Also, no structures of the components of ascosin complex were proposed.

In our studies we combined both analytical and structure elucidation methods, including HPLC-DAD-(ESI)Q-TOF and 2D NMR, to analyze and complete the available information on ascosin, candicidin and levorin aromatic heptaene antibiotic complexes in order to solve the issue regarding their similarity.

## Results and Discussion

**Figure 3:**
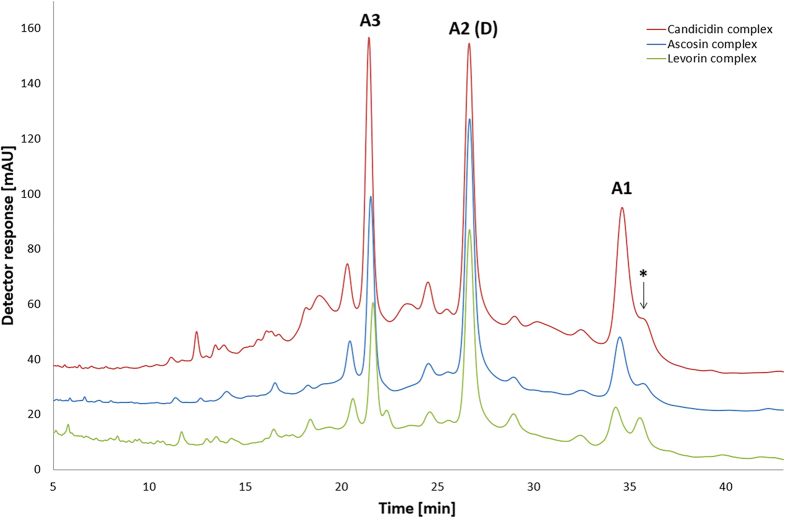
Superimposed LC-UV-VIS chromatograms of ascosin, candicidin and levorin complexes. Chromatographic conditions: column Agilent Eclipse XDB-C18, (150 mm × 4.6 mm, 5 μm). Mobile phase composition: 39% acetonitrile/61% ammonium acetate buffer (5.5 mmol, pH = 4.5), v/v; at a flow rate of 0.6 mL/min; inj. volume = 10 μL. Detection at 380 nm, room temperature. Asterisk refers to one of the products of photochemical isomerisation of the native components.

Comparative HPLC-DAD-(ESI)Q-TOF analyses were performed under identical isocratic conditions in a reversed phase mode on Agilent Eclipse XDB-C18 column (150 mm × 4.6 mm, 5 μm) using 39% acetonitrile/61% ammonium acetate buffer (5.5 mmol, pH = 4.5), v/v, solvent system. This solvent system was firstly applied in HPLC analysis of aromatic heptaenes by Y. Zhou *et al*.^26^ and then adjusted to our studies. These chromatographic conditions enabled the best known-to-date separation of the three studied complexes. The resulting chromatograms revealed that each complex is a mixture of three major aromatic heptaene antibiotics. The superimposition of the obtained chromatograms (Fig. 3) also indicated that these three complexes share a very similar elution profile and differ only in their relative composition, which depends on the particular *Streptomyces* strain used and biofermentation conditions. The individual, mutually corresponding, components of ascosin, candicidin and levorin complexes exhibit equal retention times.

The UV-VIS spectra of all the major components evidenced the presence of heptaene chromophore of *cis-trans* geometry in each case, as well as the high-resolution MS enabled the determination of their molecular masses. Basing upon the resulting *m/z* values of the ions of [M + H]^+^ type and taking into consideration the biogenetic rules, the empirical formulas have been established for all major components (Δppm < 5.1). The MS spectra obtained for each peak were in full agreement with theoretical monoisotopic mass distribution corresponding to the established formulas (Table 1).

Basing upon the aforementioned data, we assigned all the known compounds, namely candicidin D, levorin A1, levorin A2 and levorin A3, to the respective peaks. Since only in case of the levorin complex all the three major components were structurally characterized before, its nomenclature system deserves precedence and is proposed for the ascosin and candicidin complexes. Only the name of candicidin D was maintained due to its historical importance. Thus, the remaining major candicidins were named A1 and A3 and the major ascosins were named A1, A2 and A3, respectively.

The data presented in Table 2 together with the data resulting from UV-VIS spectra proved that the components of each complex with equal retention times have the same molecular masses, empirical formulas and geometry of the chromophores. Thus, it may be assumed that the three major components of ascosin, candicidin and levorin complexes are identical. Obviously, large complex molecules of equal molecular masses might be constitutional or steric isomers. Nevertheless, since retention times of corresponding components of the studied complexes are equal, structural differences between them are rather unlikely. This assumption is supported by the following structural evidence.

As it was mentioned before, the structure of the main component of the candicidin complex, candicidin D, has been evidenced to be identical with the structure of levorin A2, the main component of the levorin complex^8^. As concerns the major component of the ascosin complex, ascosin A2, herein shown to exhibit the same chromatographic and spectroscopic parameters as candicidin D and levorin A2, no structural data have been available so far. Therefore, in order to validate the resulting analytical data described above, the complete structural 2D NMR studies of this compound were performed.

It has been shown before that polyene macrolides are, in chemical terms, difficult to work with and not well soluble in the generally used NMR solvents. In order to enable the structural studies on ascosin A2, the native ascosin complex, isolated from fermentation broth of *Streptomyces canescus*, was transformed into its methyl ester of 3′-N-acetyl derivative. It has been established earlier^18^,^23^ that this type of derivatization of aromatic heptaene complexes significantly enhances their chemical properties, facilitating purification and isolation of their individual components. Also, methyl ester N-acetyl derivatives of aromatic heptaenes can be dissolved in a variety of widely used NMR solvents. This effect can be achieved in a series of simple reactions involving the usage of acetic anhydride and diazomethane as reagents.

The native ascosin complex was subjected to the reactions mentioned above. After the derivatization, the 3′-N-acetylascosin A2 methyl ester, designated as A2*, has been isolated by semi-preparative HPLC using 68% methanol/32% water, v/v, solvent system on LiChrospher 100 RP18e column (250 mm × 10 mm, 10 μm). The obtained product was a subject to NMR studies in pyridine-*d*_*5*_:methanol-*d*_*4*_ 9:1, v/v solvent system, consisting of DQF-COSY, TOCSY, edited HSQC, HMBC and ROESY experiments.

Elucidation of the structure and stereochemistry of the A2* was performed by the previously elaborated general procedure, developed via stereochemical studies of the following polyene macrolides: nystatin A1^27^, vacidin A^28^,^29^, pimaricin^30^ and amphotericin B^31^. DQF-COSY spectrum allows tracing of connectivities within isolated structural blocks separated by non-protonated carbon atoms, glycosidic bond and lactone bond. These blocks are then connected upon the HMBC experiment revealing long range heteronuclear couplings. The geometry of polyene chromophore is revealed by vicinal coupling constants, UV-VIS spectrum and chemical shifts of resonances observed in ^13^C NMR spectrum. The next step is the assignment of the relative configurations of stereogenic centers within the macrolactone ring by analysis of vicinal coupling constants and appropriate ROEs/NOEs. Having the relative configuration of the aglycone unit in hand, one can exploit glycosidically bound and stereochemically defined monosaccharide moiety (which is a D-mycosamine moiety in most cases) as a naturally occurring internal chiral probe. The absolute configuration of the aglycone stereogenic center linked via glycosidic bond with monosaccharide moiety is pointed out by the appropriate ROEs between the protons of the macrolactone unit and the protons of the chiral probe.

The full ^1^H and ^13^C assignments for A2* are given in Tables 3 and 4, respectively, and the complete stereostructure of A2* was derived as follows.

Basing upon the ^13^C chemical shift values and HSQC & HMBC spectra, the C2-C14 hydrophilic fragment was proven to contain two carboxyl groups in positions C3 and C7 and three hydroxyl groups in positions C9, C11 and C13, with the remaining positions established to be the methylene groups via edited HSQC spectrum. The relative configuration of the C9, C11 and C13 carbon atoms was established as (9R*, 11S*, 13S*) due to the H9-H11-H13 ROE pathway, the ROEs H9/H26, H11/H24, H11/H26, H13/H22, H13/H24 and the ^3^J_H,H_ coupling constants within the C8-C14 structural block (Table 3). The C15-C19 fragment was revealed to be a six-membered ring with oxygen bridge between C15 and C19. This was evidenced by the characteristic hemiketal carbon chemical shift of C15 (δ = 98.0 ppm) and the HMBC correlation H19-C15. The analysis of vicinal coupling constants within this fragment, combined with ROEs H14a/H16a, H14b/H16b, H18/H20a, H19/H22 and H21/H23 pointed out the relative configuration of C15, C17, C18, C19 and C21 stereogenic centers as (15R*, 17S*, 18R*, 19S*, 21R*). HMBC and ROESY spectra also revealed the presence of the D-mycosamine moiety, β-glycosidically bound to the C21 carbon atom of the macrolactone ring.

The data extracted from DQF-COSY and ROESY spectra confirmed the presence of C22-C35 heptaene chromophore, postulated earlier by the UV-VIS data. The geometry of the chromophore was established as 22E, 24E, 26Z, 28Z, 30E, 32E and 34E and was derived from a set of vicinal coupling constants (Table 3). While most of the ^3^J_H,H_ coupling constants within the chromophore contained in a range from 15 to 16 Hz and determined the E geometry, the ^3^J_26,27_ and ^3^J_28,29_ were equal to 11 Hz and indicated to the presence of two double bonds with the Z geometry. This assignment was strongly supported by the presence of the ROEs H25/H28, H26/H27, H27/H30 and H28/H29.

The structure of the C37-attached sidechain was established upon all the 2D NMR experiments conducted in this study. It was revealed to consist of a linear six carbon skeleton with several substituents attached, ending with p-aminoacetophenone moiety. Analysis of the vicinal coupling constants and the appropriate ROEs within the sidechain pointed out the relative configuration of the C36-C42 fragment as (36S*, 37R*, 38S*, 40S*, 41S*). The configuration of the C36 and C37 stereogenic centers in relation to the C8-C21 fragment was deduced upon the fact that only one of two enantiomers possible for closing the macrolactone ring was in full agreement with the spectral data. If the relative configurations of C36 and C37 were opposite, it would imply the crossing of the chromophore and the polyol chains, which would have resulted in ROE contacts between the C4-C6 fragment and the chromophore. Such correlations have not been observed. Moreover, aforesaid conformation of the macrolactone would disrupt the regular alignment of the C9-C13 fragment, which was clearly evidenced by the NMR data (Table 3).

The ROESY spectrum revealed the presence of several dipolar couplings between the protons of the aglycone unit and the protons of the D-mycosamine moiety. These correlations, namely H1′/H20b, H1′/H21 and H2′/H19, unambiguously pointed out the absolute configuration of C21 as (21R). Therefore, the absolute configuration of all the stereogenic centers of the aglycone unit was established as (9R, 11S, 13S, 15R, 17S, 18R, 19S, 21R, 36S, 37R, 38S, 40S, 41S).

In the end, the structure of the 3′-N-acetylascosin A2 methyl ester (A2*), resulting from the 2D NMR data, proved that the structure of ascosin A2 is identical with structures of the levorin A2 and the candicidin D. Also, due to the results presented above, the absolute configurations of all the stereogenic centers of candicidin D and levorin A2 have also been defined.

## Conclusions

**Figure 4:**
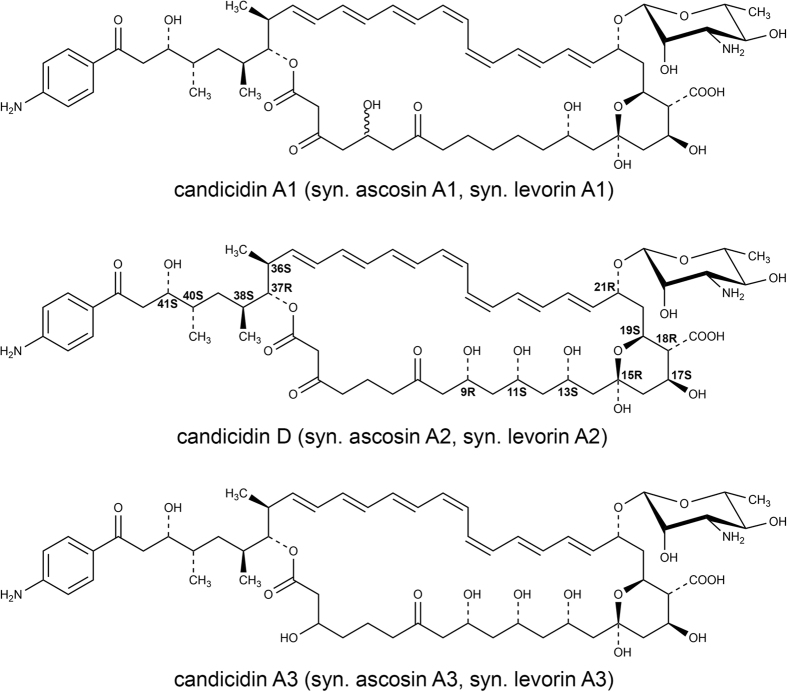
The structures of the three major antibiotics of the ascosin, candicidin and levorin complexes.

In this work, we presented novel structural data, supplemented with comparative analytical studies, which have evidenced that ascosin, candicidin and levorin are complexes of three identical major polyene macrolide antibiotics of aromatic heptaene group: A1, A2 (D) and A3 respectively, presented in Fig. 4, as well as several minor components. These complexes differ only by the relative amount of their components. Therefore, the structures of candicidin A1 and A3 and ascosin A1 and A3 with partially defined stereochemistry should be assumed as known as well as the stereostructure of candicidin D and levorin A2. Furthermore, because candicidin complex was the first of these antibiotics to be reported in the literature, its name deserves precedence and the names ascosin and levorin should be used only as synonyms.

## Methods

### Samples of aromatic heptaene macrolide antibiotics

#### Ascosin complex

The crude ascosin complex was obtained by extraction with n-butanol from fermentative broth of *Streptomyces canescus* in the Department of Pharmaceutical Technology and Biochemistry, Gdańsk University of Technology (Gdańsk, Poland).

#### Candicidin complex

The crude candicidin complex was supplied by Tarchomin Pharmaceutical Industry ‘Polfa’ (Warsaw, Poland).

#### Levorin complex

The sample of levorin complex was a kind gift of dr. Yu. Shenin from the All Union Institute of Antibiotics and Enzymes of Medical Use, Petersburg (former Leningrad), Russia.

### Synthesis of 3′-N-acetylascosin A2 methyl ester (A2*)

The crude ascosin complex was N-acetylated by the following procedure. To suspension of 200 mg of ascosin complex in 20 ml of solvent mixture MeOH - H_2_O, 9:1 (v/v), 80 μl of acetic anhydride was added. The reaction was carried out at room temperature and its progress was monitored by TLC on Si 60, Merck and solvent system EtOAc - AcOH - H_2_O, 4:1:1 (v/v/v). After 30 minutes the reaction mixture was centrifuged, 5 ml of *n*-butanol was added to the supernatant and a volume of the resulting solution was reduced to about 1 ml by evaporation under reduced pressure. The product was precipitated from *n*-butanol solution with 30 ml of dry ethyl ether and centrifuged. The precipitate was washed three times with 30 ml of dry ethyl ether and dried under reduced pressure. Yield 70 mg of N-acetylated ascosin complex. The procedure was repeated several times to obtain the required amount of final product.

5.3 g of 3′-N-acetylascosin complex was obtained and it was further purified by flash chromatography on Kieselgel Si 60, 0.063–0.200 mm, Merck and solvent system CHCl_3_ - MeOH - H_2_O, 5:2:0.2 (v/v/v). 480 mg of 3′-N-acetylascosin complex, 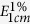 = 644 (380 nm), was dissolved in 150 ml of solvent mixture MeOH - H_2_O, 9:1 (v/v) and diazomethane in ethyl ether was added dropwise. The reaction was carried out at room temperature and its progress was monitored by TLC on Si 60, Merck and solvent system EtOAc - AcOH - H_2_O, 4:1:1 (v/v/v). After further addition of 35 ml of *n*-butanol, methanol and water were evaporated under reduced pressure. The derivatized antibiotic complex was then precipitated from *n*-butanol with 120 ml of dry ethyl ether and centrifuged. The precipitate was washed three times with 50 ml of dry ethyl ether and dried under reduced pressure. Yield 320 mg, 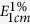 = 600 (380 nm). The flash chromatography on Kieselgel Si 60, 0.063–0.200 mm, Merck and solvent mixture CHCl_3_ - MeOH - H_2_O, 5:0.75:0.075 (v/v/v) was performed to separate the product from non-esterified antibiotics. Yield 110 mg, 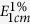 = 966 (380 nm).

The isolation of 3′-N-acetylascosin A2 methyl ester (A2*) from 110 mg of the derivatized antibiotic complex was performed by means of HPLC on a Merck-Hitachi apparatus L-6200 A, equipped with Merck-Hitachi L-4250 UV-VIS detector. The separation conditions were as follows: column LiChrospher 100 RP-18e (250 mm × 10 mm, 10 μm). Mobile phase composition: 68% Me OH/32% H2O, v/v; at a flow rate of 6.25 ml/min. Detection at 380 nm, room temperature. 8.25 mg of the sample dissolved in 625 μl of mixture MeOH - H_2_O, 95:5 (v/v) was injected. The retention time of the 3′-N-acetylascosin A2 methyl ester (A2*) was 43 min. HPLC separation was performed 12 times giving 11 mg of A2* as yellow-green powder. 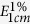 = 990 (380 nm).

### NMR experimental

The NMR spectra were recorded with a Bruker Ascend 700 MHz spectrometer in solvent system pyridine-*d*_*5*_—methanol-*d*_*4*_, 9:1 (v/v) with a sample concentration of 10 mg ml^−1^.

One-dimensional ^1^H spectra were collected using standard parameters.

Two-dimensional ^1^H spectra were measured in the phase-sensitive mode with a spectral width of 7716 Hz. The DQF-COSY spectrum was acquired in a 6080 × 512 matrix with 32 accumulations per increment and was processed in a 4 K×2 K matrix. The TOCSY spectrum was acquired with a mix time of 110 ms in a 2048 × 512 matrix with 32 accumulations per increment in a 2 K × 1 K matrix. The ROESY spectrum was acquired with a mix time of 300 ms in a 2048 × 512 matrix with 64 accumulations per increment in a 2 K × 1 K matrix. HSQC and HMBC experiments were performed with pulse field gradients. The HSQC spectrum was acquired in the phase-sensitive mode. The spectral windows for ^1^H and ^13^C axes were 7716 Hz and 35211 Hz, respectively. The data were collected in a 1024×256 matrix and processed in a 2 K × 1 K matrix. The HMBC spectrum was acquired in absolute value mode. The spectral windows for ^1^H and ^13^C axes were 7716 Hz and 39063 Hz, respectively. The data were collected in a 2048×256 matrix and processed in a 2 K × 1 K matrix.

### LC-MS experimental

High-resolution MS spectra were recorded with Agilent Technologies 6540 UHD Accurate-Mass Q-TOF spectrometer in positive electrospray ionization mode. The chromatographic separation was performed using the chromatographic system consisting of a pump, degasser, autosampler and column oven from the Agilent 1290 Infinity series. The separation of analytes was achieved using a Agilent Eclipse XDB-C18 column (150 mm × 4.6 mm, 5 μm, pore size 80 Å). The mobile phase contained 39% acetonitrile/61% ammonium acetate buffer (5.5 mmol, pH = 4.5), v/v; at a flow rate of 0.6 mL/min.

## Additional Information

**How to cite this article:** Szczeblewski, P. *et al*. Analytical studies on ascosin, candicidin and levorin multicomponent antifungal antibiotic complexes. The stereostructure of ascosin A2. *Sci. Rep.* **7**, 40158; doi: 10.1038/srep40158 (2017).

**Publisher's note:** Springer Nature remains neutral with regard to jurisdictional claims in published maps and institutional affiliations.

## Supplementary Material

Supplementary Information

## Figures and Tables

**Figure 1:**
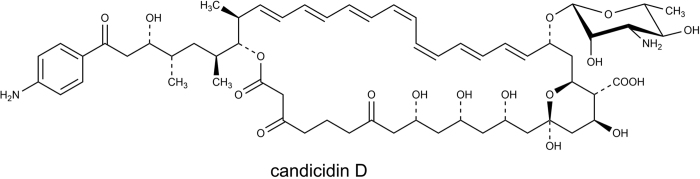
The structure of candicidin D, the main component of the candicidin complex.

**Figure 2:**
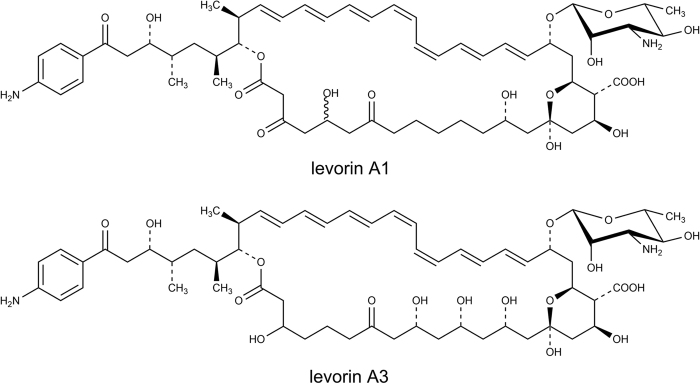
The structures of levorin A1 and A3 with partially defined stereochemistry.

**Table 1:** The resulting *m/z* values of the [M + H]^+^ ions of the major components of the studied complexes.

Retention time of the major component [min]	Molecular formula	Monoisotopic mass calculated for ion of [M + H]^+^ type	Empirical monoisotopic mass obtained for ion of [M + H]^+^ type					
			Ascosin		Candicidin		Levorin	
			[M + H]^+^	∆ [ppm]	[M + H]^+^	∆ [ppm]	[M + H]^+^	∆ [ppm]
21.4	C_59_H_86_O_18_N_2_	1111.59479	1111.5933	1.49	1111.5930	1.79	1111.5926	2.19
26.6	C_59_H_84_O_18_N_2_	1109.57914	1109.5788	0.34	1109.5789	0.24	1109.5763	2.84
34.4	C_59_H_84_O_17_N_2_	1093.58423	1093.5838	0.43	1093.5822	2.03	1093.5792	5.03

**Table 2:** The composition of the ascosin, candicidin and levorin antibiotic complexes.

Component of the complex		A3	A2 (D)	A1
Molecular formula		C_59_H_86_O_18_N_2_	C_59_H_84_O_18_N_2_	C_59_H_84_O_17_N_2_
Monoisotopic molecular mass [u]		1110.59	1108.57	1092.58
Retention time [min]		21.4	26.6	34.4
Contribution [%]	Ascosin	20%	41%	12%
	Candicidin	21%	28%	15%
	Levorin	17%	37%	11%

**Table 3:** ^1^H NMR data for 3′-N-acetylascosin A2 methyl ester (A2*).

Proton no.	^1^H δ (p.p.m.)	J_H,H_ (Hz)	ROE contacts
**Aglycone**			
2a	3.59	14.3 (2b)	2b
2b	3.68	14.3 (2a)	2a
4a	2.54	15.2 (4b), 5.3 (5)	5, 4b
4b	2.63	15.2 (4a), 10.7 (5)	4a
5 (2H)	1.92	5.3 (4a), 10.7 (4b), 7.6 (6)	4a, 6
6 (2H)	2.42	7.6 (5)	5
8a	2.43	14.5 (8b), 4.2 (9)	8b, 9, 10a
8b	2.72	14.5 (8a), 9.0 (9)	8a, 10b, 11
9	4.65	4.2 (8a), 9.0 (8b), 3.6 (10a), 8.4 (10b)	8a, 10a, 11, 26
10a	1.53	3.6 (9), 13.7 (10b), 3.6 (11)	8a, 9, 10b, 11
10b	1.77	8.4 (9), 13.7 (10a), 9.1 (11)	8b, 10a, 11
11	4.27	3.6 (10a), 9.1 (10b), 4.0 (12a), 9.1 (12b)	8b, 9, 10a, 10b, 13, 24, 26
12a	1.52	4.0 (11), 14.7 (12b), 3.5 (13)	12b, 13
12b	1.79	9.1 (11), 14.7 (12a), 10.3 (13)	12a, 14b
13	4.75	3.5 (12a), 10.1 (12b), 3.5 (14a), 10.3 (14b)	11, 12a, 14a, 22, 24
14a	1.80	14.8 (14b), 3.5 (13)	13, 14b, 16a
14b	2.00	14.8 (14a), 10.1 (13)	12b, 14a, 16b
16a	1.78	13.5 (16b), 8.3 (17)	14a, 18, 16b
16b	2.55	13.5 (16a), 4.6 (17)	14b, 17, 16a
17	5.02	8.3 (16a), 4.6 (16b), 10.5 (18)	16b
18	2.85	10.5 (17), 10.5 (19)	16a, 19, 20a, 21
19	5.00	10.5 (18), 6.4 (20a), 2.3 (20b)	18, 2’
20a	2.11	6.4 (19), 14.9 (20b), 1.3 (21)	18, 20b, 21
20b	2.38	2.3 (19), 14.9 (20a), 5.1 (21)	20a, 21, 1′
21	4.93	1.3 (20a), 5.1 (20b), 7.5 (22)	18, 20a, 20b, 22, 23, 1’
22	6.37	7.5 (21), 15.0 (23)	13, 21, 24
23	6.47	15.0 (22),11.0 (24)	21, 25
24	6.64	11.0 (23), 16.0 (25)	11, 13, 22, 26
25	6.94	16.0 (24), 11.0 (26)	23, 28
26	6.46	11.0 (25), 11.0 (27)	9, 11, 24, 27
27	6.93	11.0 (26), 11.0 (28)	26, 30
28	6.75	11.0 (27), 11.0 (29)	25, 29
29	6.32	11.0 (28), 11.0 (30)	28, 31
30	7.18	11.0 (29), 16.0 (31)	27, 32
31	6.47	16.0 (30), 11.0 (32)	29
32	6.55	11.0 (31), 15.0 (33)	30, 34
33	6.40	15.0 (32), 10.0 (34)	35
34	6.23	10.0 (33), 15.0 (35)	32, 36
35	5.64	15.3 (34), 9.0 (36)	33, 36, 37, Me36
36	2.57	9.0 (35), 6.5 (Me36), 9.7 (37)	34, 35, Me36
37	5.06	9.7 (36), 3.3 (38)	35, 38, 39a, Me36
38	2.02	3.3 (37), 6.9 (Me38), 4.2 (39a), 7.6 (39b)	37, Me38
39a	1.37	4.2 (38), 16.3 (39b), 11.1 (40)	37, 39b, Me38
39b	1.79	7.6 (38),16.3 (39a), 4.0 (40),	39a, 40, 41, 42a, Me40
40	2.00	11.1 (39a), 4.0 (39b), 7.3 (Me40), 8.7 (41)	39b, 41, 42b, Me40
41	4.52	8.7 (40), 4.5 (42a), 8.4 (42b)	39b, 40, 42a, 42b, Me38, B
42a	3.15	4.5 (41), 15.6 (42b)	39b, 41, 42b, B
42b	3.40	8.4 (41), 15.6 (42a)	40, 41, 42b, B
Me36	0.99	6.5 (36)	35, 36, 37
Me38	1.12	6.9 (38)	38, 39a, 41
Me40	1.01	7.3 (40)	39b, 40
COOMe	3.79	—	6′
**Aromatic protons**			
A (2H)	6.97	8.7 (B)	B
B (2H)	8.16	8.7 (A)	41, 42a, 42b, A
**3′-N-acetylmycosamine moiety**			
1′	4.99	1.0 (2′)	2′, 3′, 5′, 20b, 21
2′	4.45	1.0 (1′), 4.2 (3′)	1′, 3′, 19
3′	4.69	4.2 (2′), 10.0 (4′)	1′, 2′, 5′
4′	4.05	10.0 (3′), 9.4 (5′)	5′, 6′, NH
5′	3.77	9.4 (4′), 6.7 (6′)	1′, 3′, 4′, 6′
6′	1.59	6.7 (5′)	4′, 5′, COOMe
NH	8.92	—	4′, COMe
COMe	2.11	—	NH

**Table 4:** ^13^C NMR data for 3′-N-acetylascosin A2 methyl ester (A2*).

^13^C atom no.	^13^C δ (p.p.m.)	^13^C atom no.	^13^C δ (p.p.m.)
**Aglycone**		**32**	132.9
1	166.9	**33**	133.9
2	49.0	**34**	132.2
3	202.0	**35**	137.9
4	42.5	**36**	40.3
5	17.5	**37**	80.9
6	43.0	**38**	31.2
7	209.4	**39**	37.4
8	50.6	**40**	37.0
9	56.0	**41**	71.5
10	43.9	**42**	42.1
11	72.4	**43**	198.0
12	44.1	**Me36**	16.5
13	68.9	**Me38**	15.7
14	46.9	**Me40**	12.3
15	98.0	**C**OOMe	173.9
16	45.0	COO**Me**	51.7
17	66.2	**Aromatic carbons**	
18	58.6	**CA**	113.2
19	68.1	**CB**	131.3
20	37.7	C*CO	154.2
21	76.1	C*NH2	126.7
22	137.5		
23	131.5	**3′-N-acetylmycosamine moiety**	
24	134.7	**1′**	98.5
25	127.9	**2′**	70.8
26	132.9	**3′**	55.9
27	125.1	**4′**	72.2
28	124.9	**5′**	74.8
29	130.3	**6′**	18.4
30	132.4	NH**C**OMe	170.8
31	134.2	NHCO**Me**	22.8
