# Supplementary material for: Analytical studies on ascosin, candicidin and levorin multicomponent antifungal antibiotic complexes. The stereostructure of ascosin A2
Source: Sci Rep. 2017 Jan 9;7:40158. doi: 10.1038/srep40158 (PMC5220287; doi:10.1038/srep40158)
Supplement: Supplementary Information [file srep40158-s1.pdf]

Analytical studies on ascosin, candicidin and levorin multicomponent antifungal antibiotic complexes. The stereostructure of ascosin A2

Paweł Szczeblewski, Tomasz Laskowski, Bartosz Kubacki, Marta Dziergowska, Magda Liczmańska, Jakub Grynda, Paweł Kubica, Agata Kot-Wasik and Edward Borowski

ELECTRONIC SUPPLEMENTARY INFORMATION

Table of contents:

1. NMR spectra of 3’-N-acetylascosin A2 methyl ester (**A2***) 2
2. MS and UV-VIS spectra 8
2.1. UV-VIS spectra 8
2.2. MS spectra 8

1. NMR SPECTRA OF 3'-N-ACETYLASCOSIN A2 METHYL ESTER (A2*).

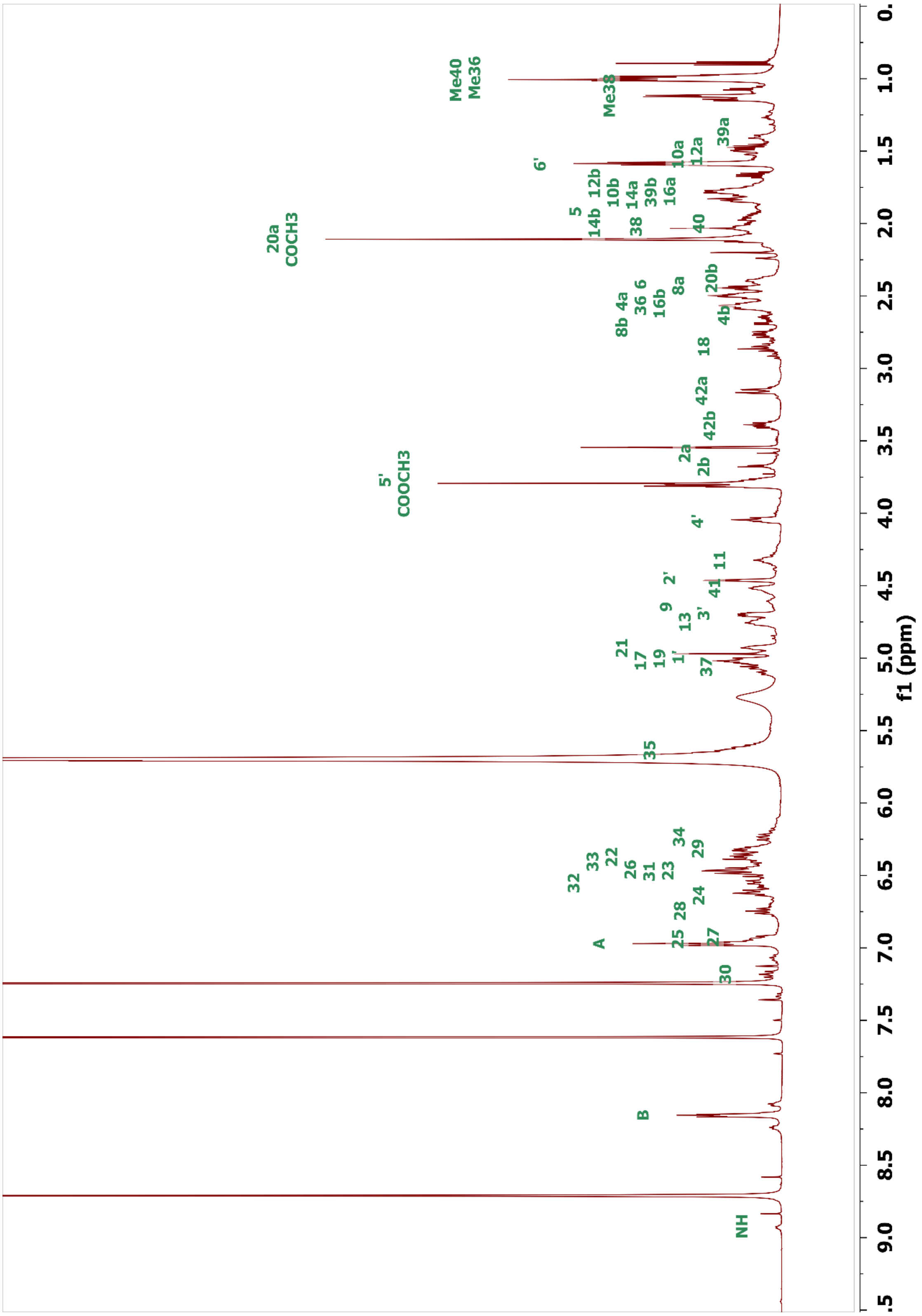

Figure 1.1. ^{1}H NMR spectrum of **A2***.

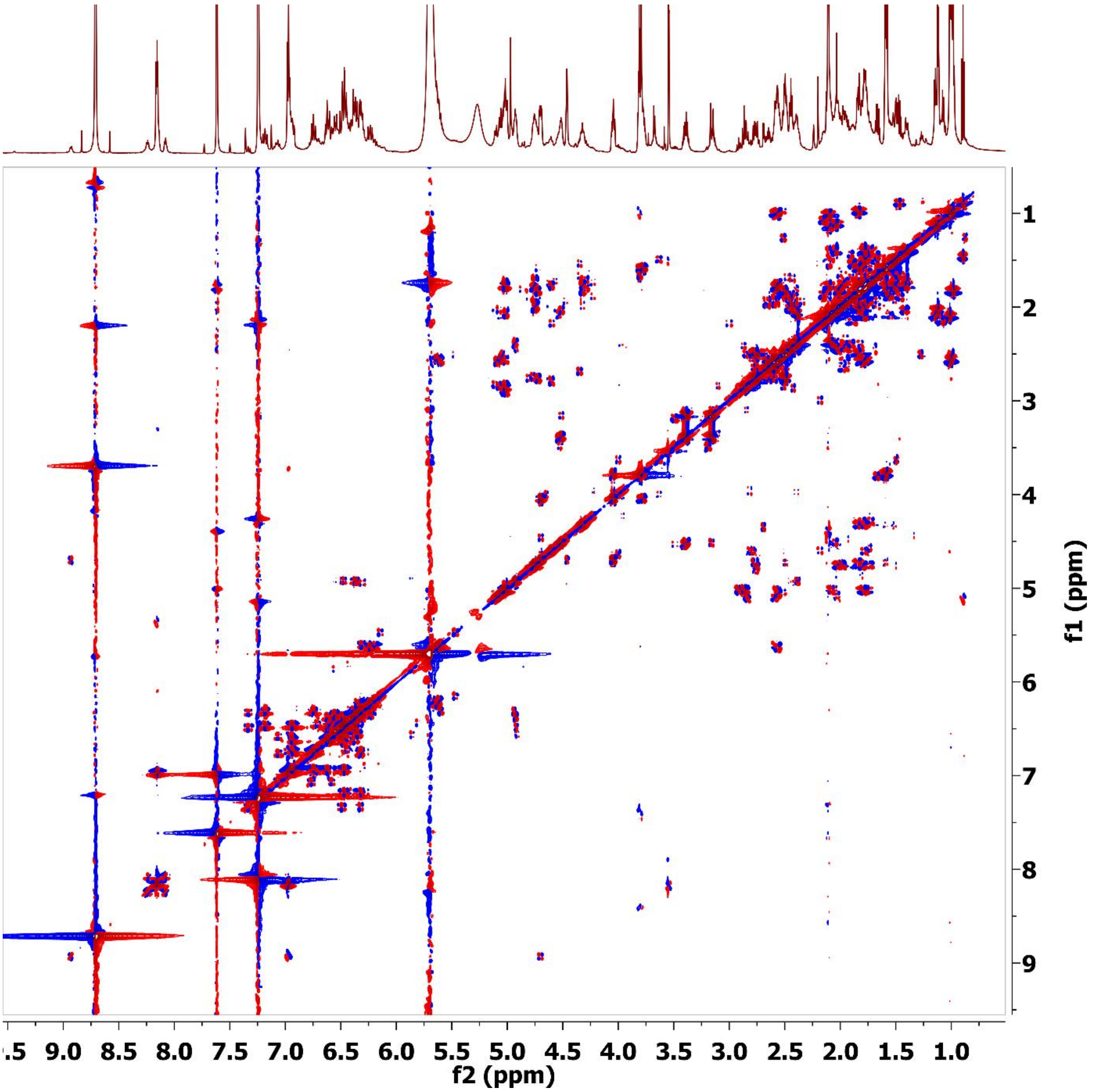

Figure 1.2. DQF-COSY spectrum of **A2***.

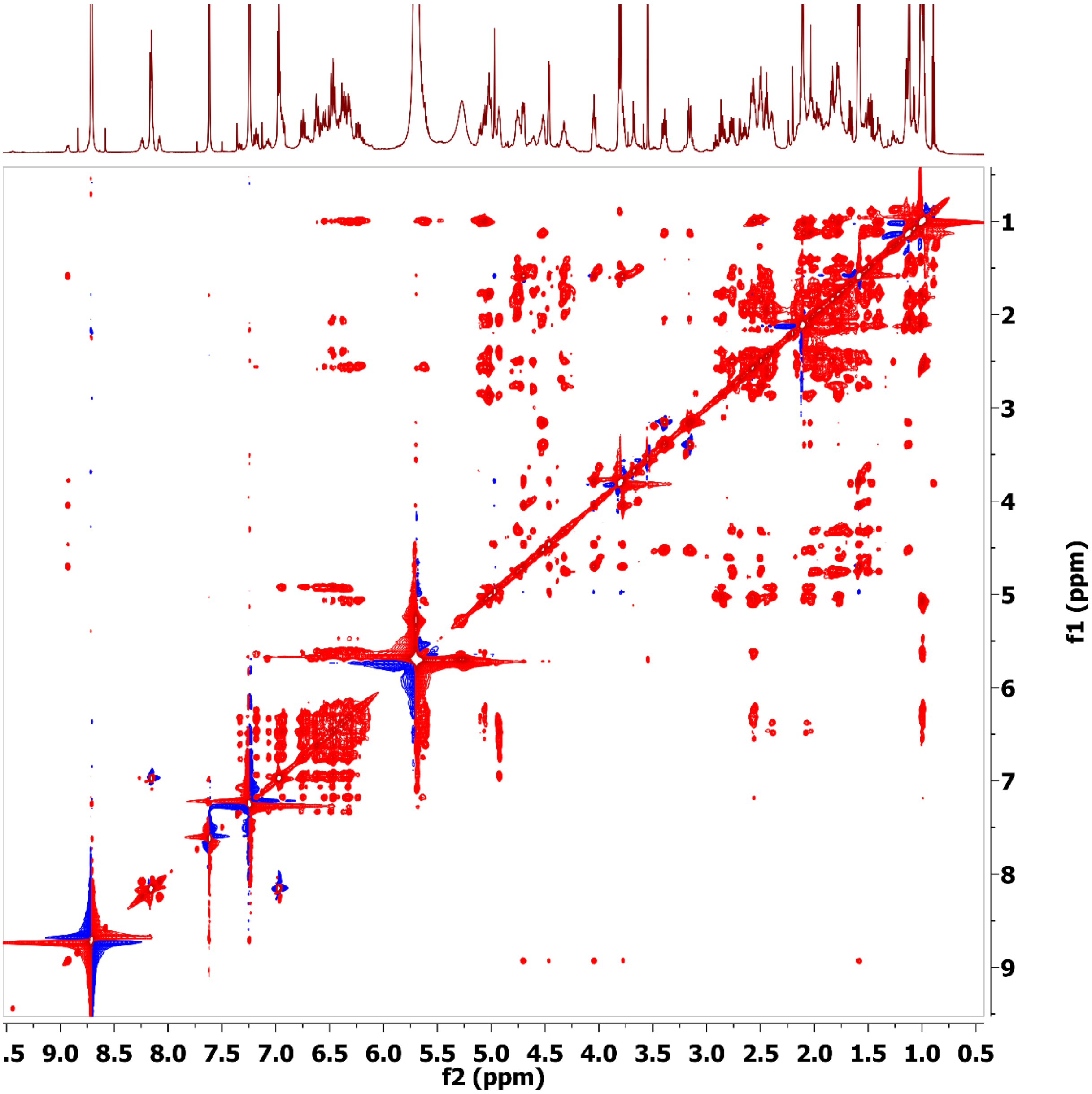

Figure 1.3. TOCSY spectrum of **A2***. Mix time = 110 ms.

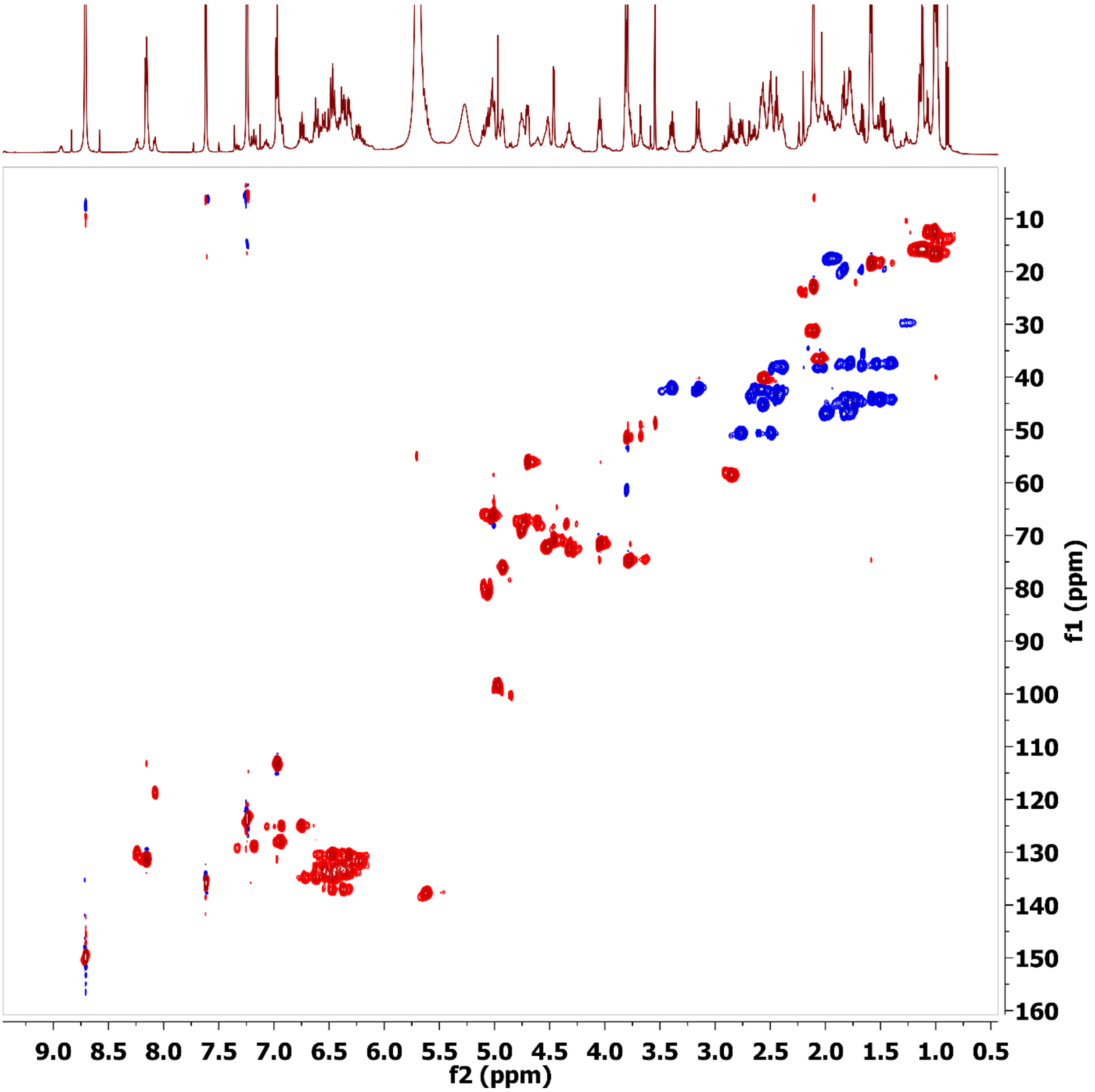

Figure 1.4. Edited HSQC spectrum of **A2***.

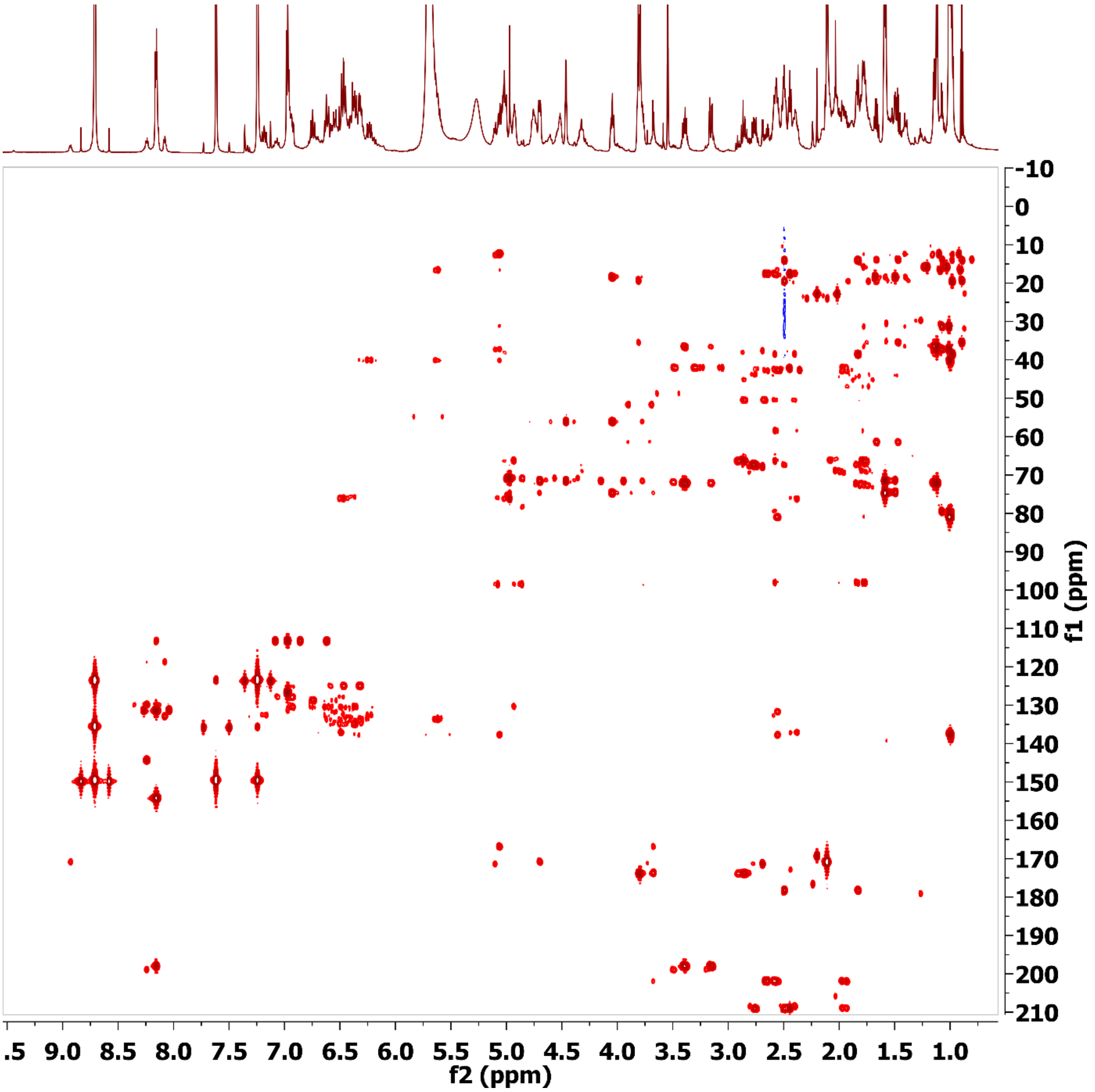

Figure 1.5. GHMBC spectrum of **A2***.

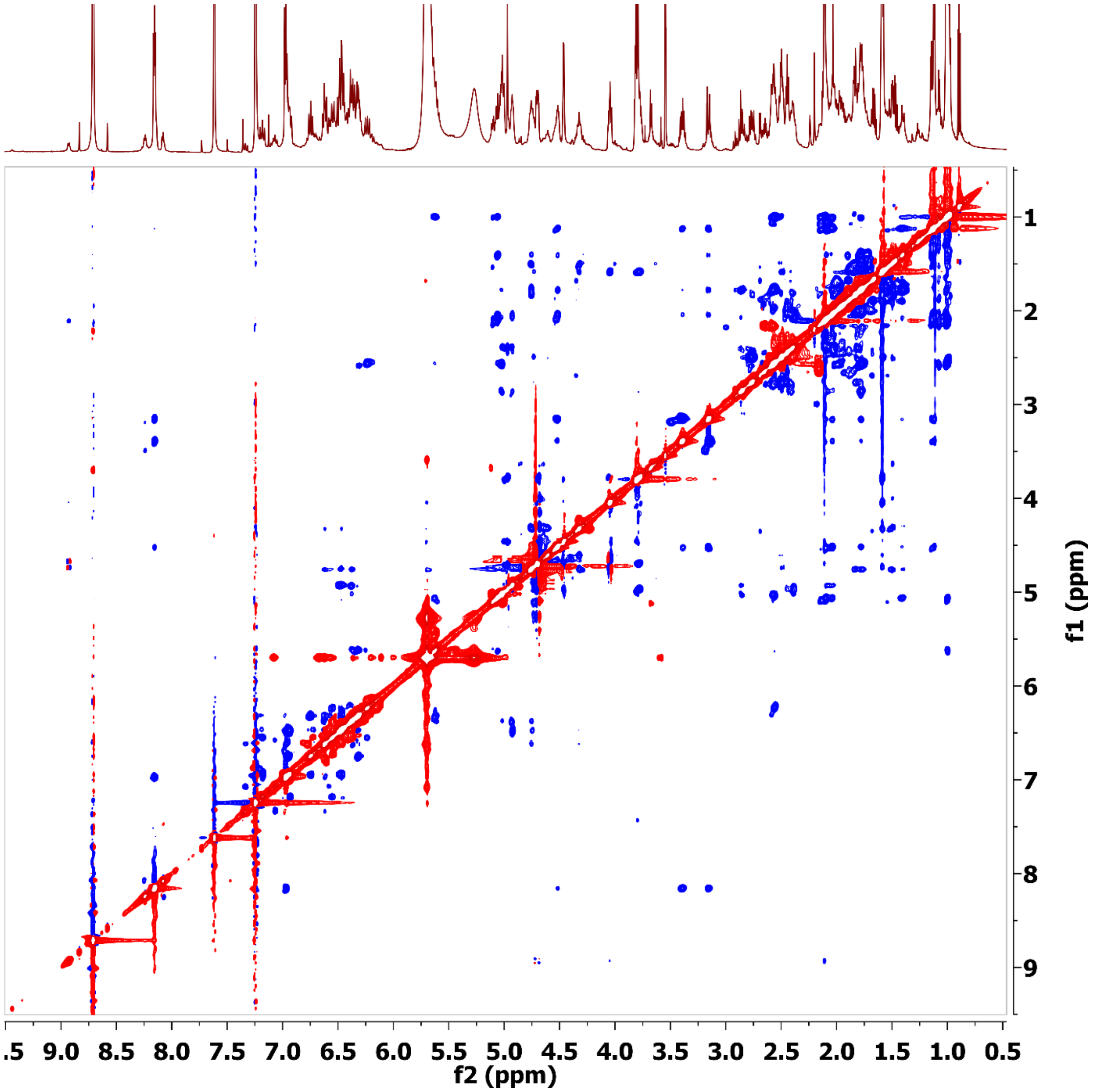

Figure 1.6. ROESY spectrum of **A2***. Mix time = 300 ms.

2. MS AND UV-VIS SPECTRA.

2.1. UV-VIS spectra.

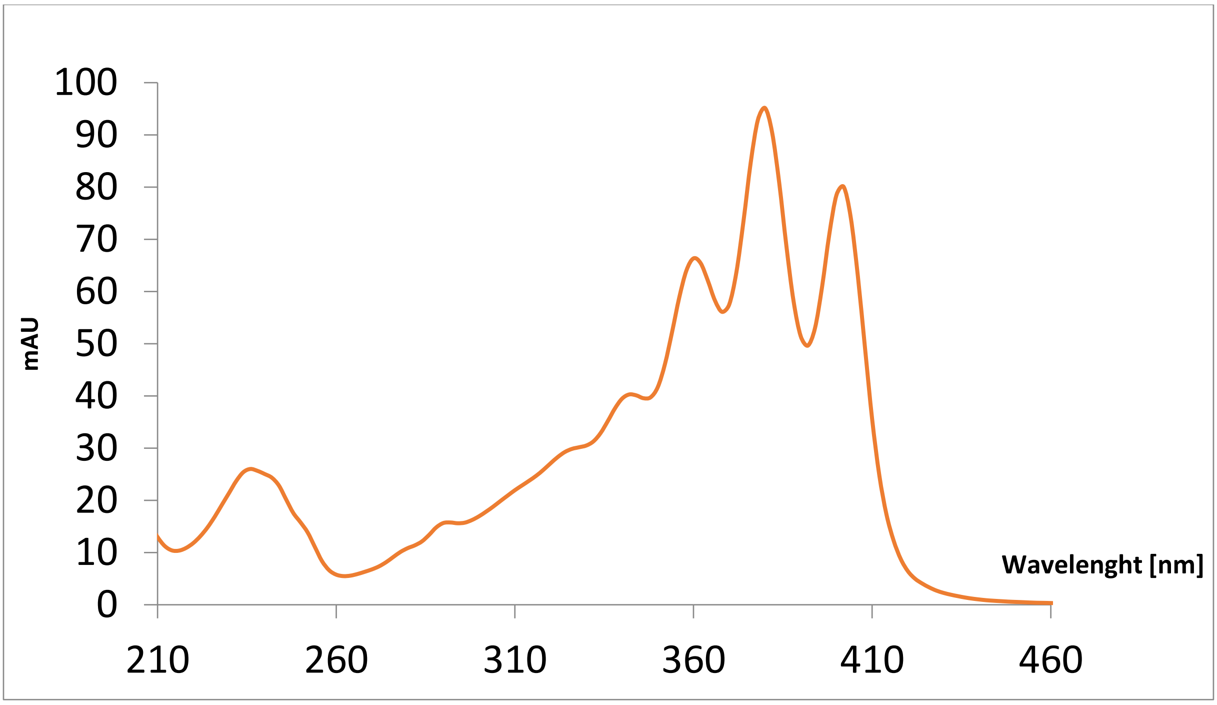

Figure 2.1. The example UV-VIS spectrum of candicidin D recorded with DAD detector. The same UV-Vis spectra were obtained for all major components of studied complexes.

2.2. MS spectra.

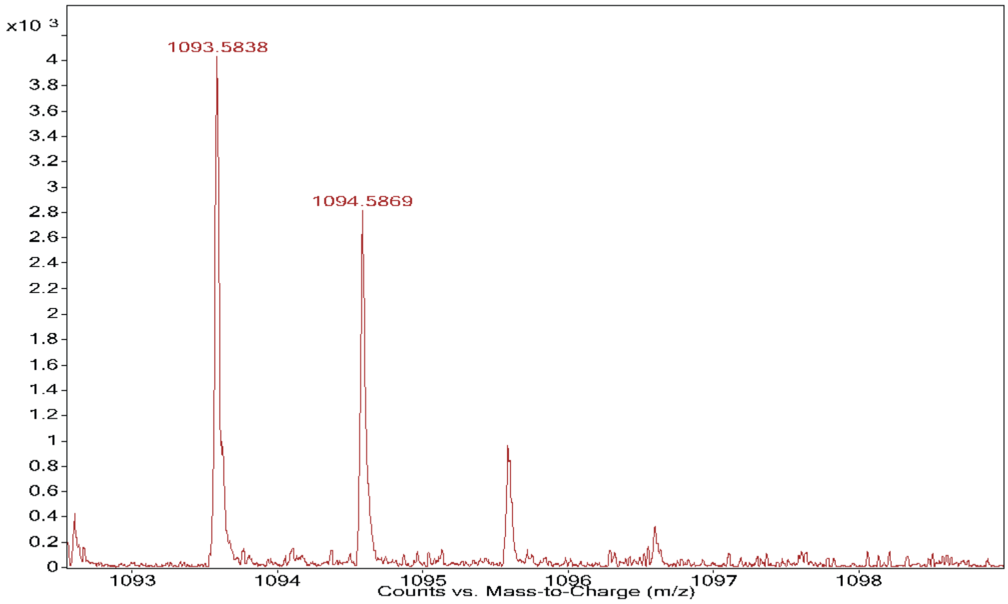

Figure 2.2. The high-resolution isotopic mass distribution of pseudomolecular ions $[M+H]^+$ of ascosin A1.

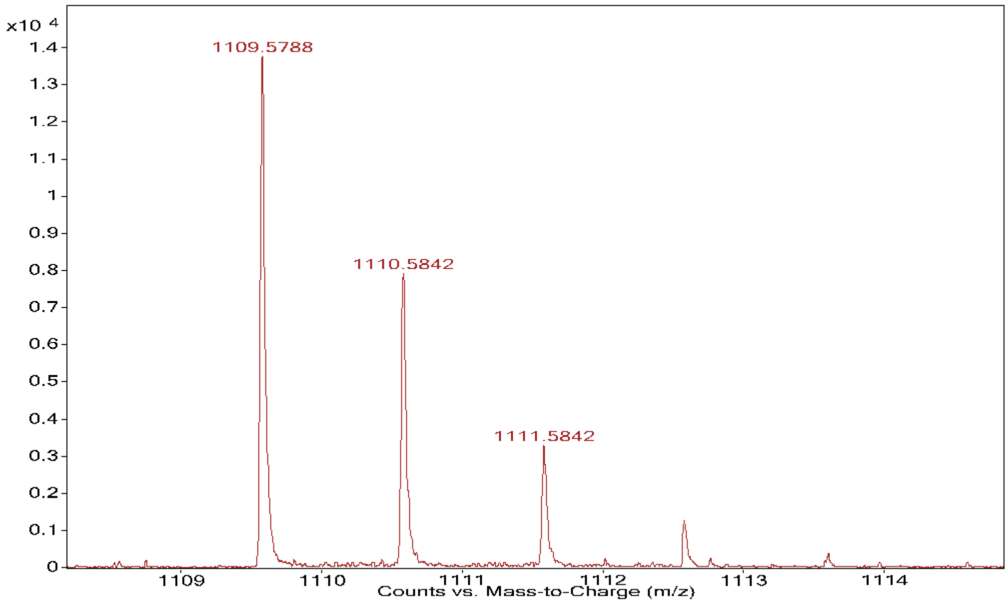

Figure 2.3. The high-resolution isotopic mass distribution of pseudomolecular ions $[M+H]^+$ of ascosin A2.

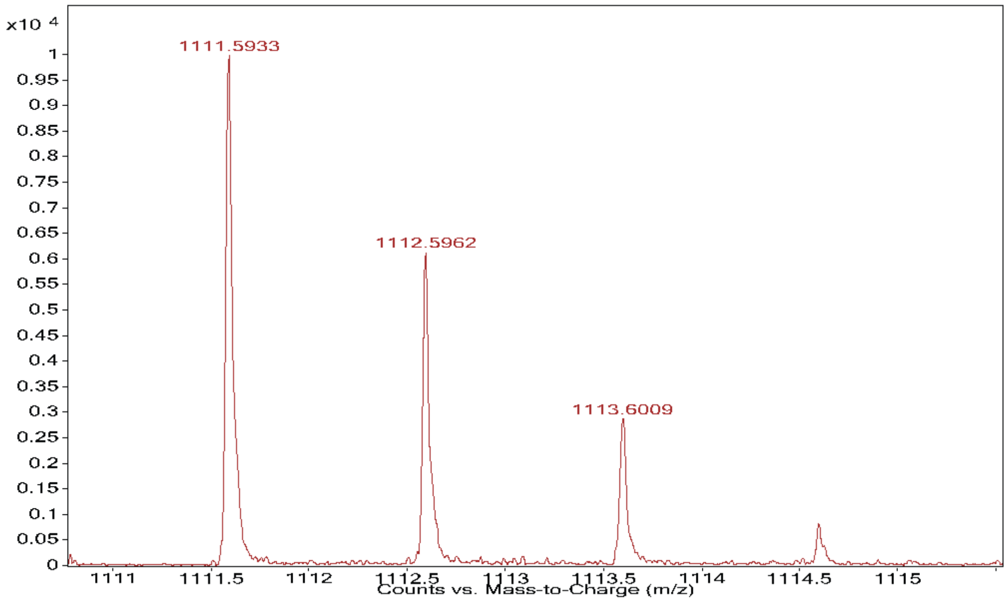

Figure 2.4. The high-resolution isotopic mass distribution of pseudomolecular ions $[M+H]^+$ of ascosin A3.

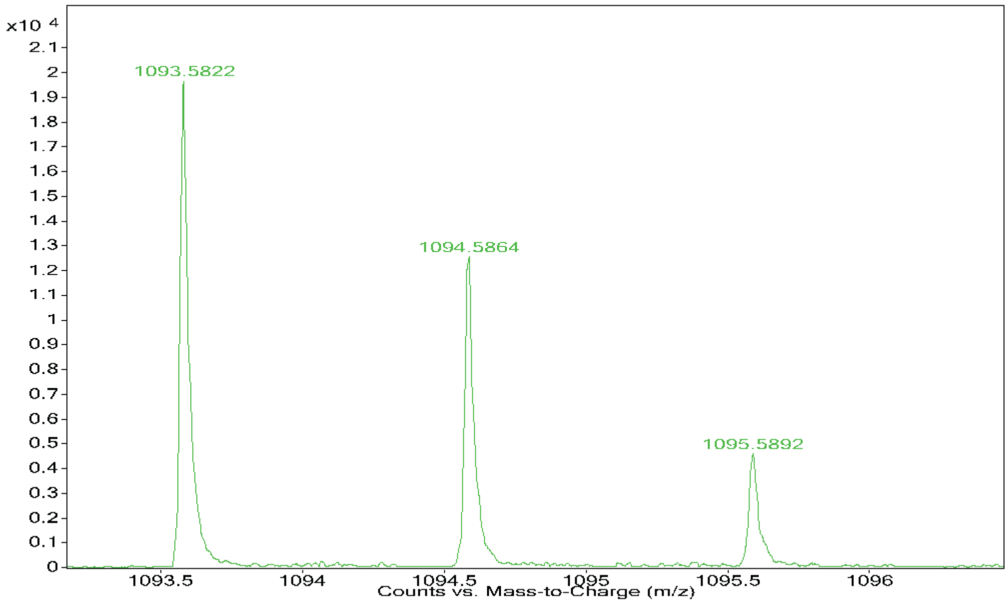

Figure 2.5. The high-resolution isotopic mass distribution of pseudomolecular ions $[M+H]^+$ of candicidin A1.

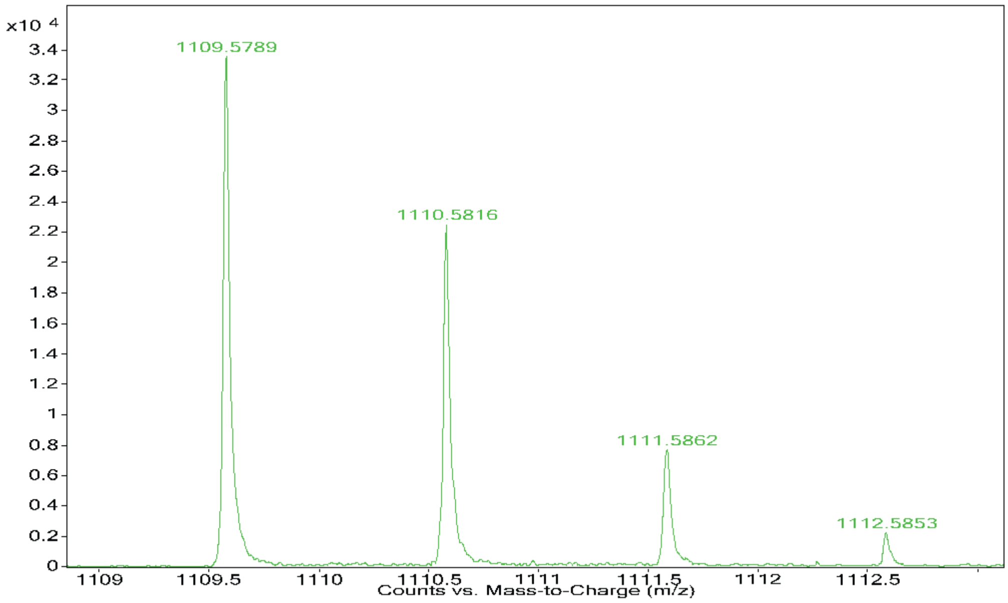

Figure 2.6. The high-resolution isotopic mass distribution of pseudomolecular ions $[M+H]^+$ of candicidin D.

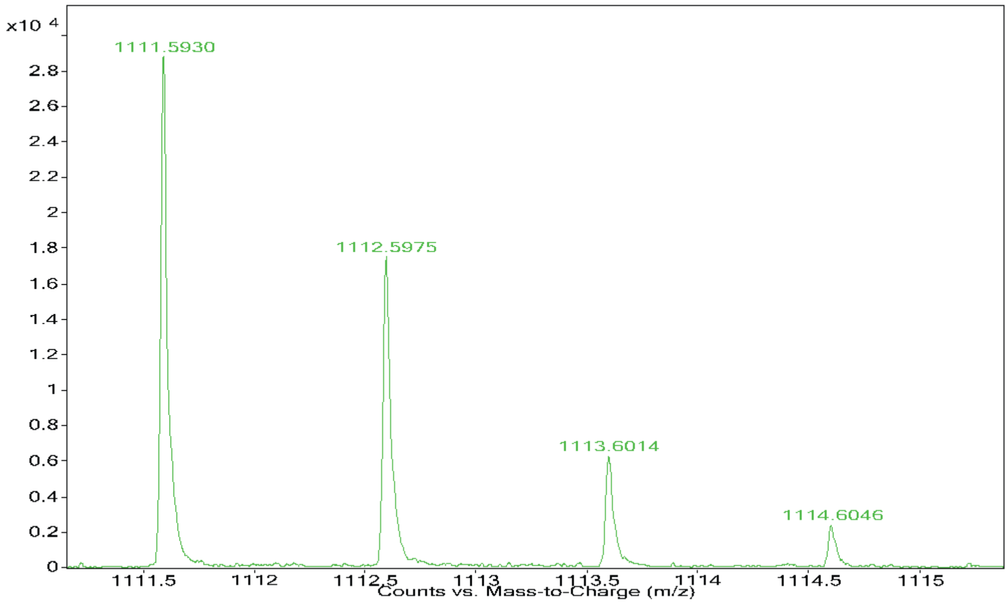

Figure 2.7. The high-resolution isotopic mass distribution of pseudomolecular ions $[M+H]^+$ of candicidin A3.

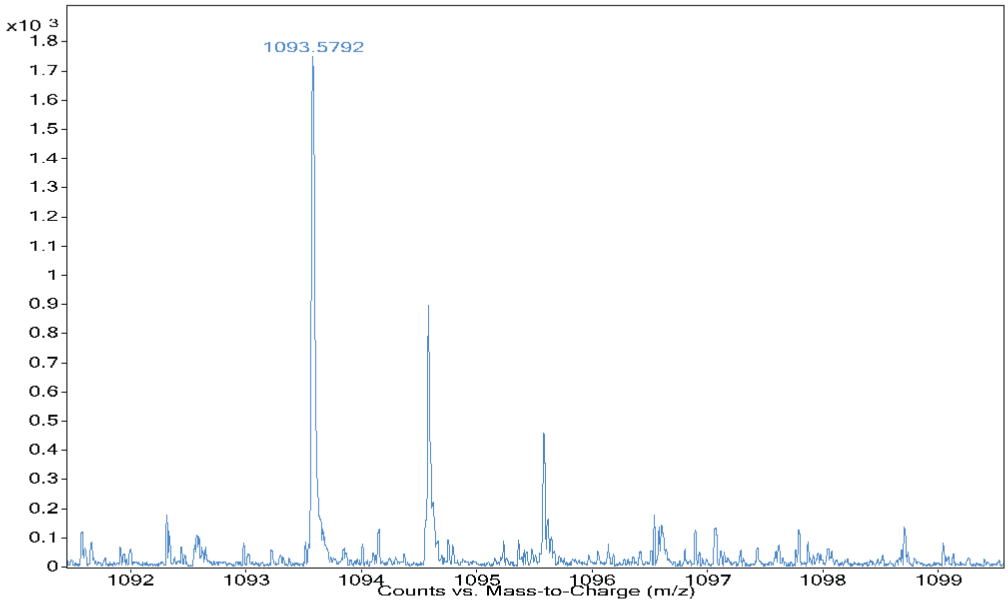

Figure 2.8. The high-resolution isotopic mass distribution of pseudomolecular ions $[M+H]^+$ of levorin A1.

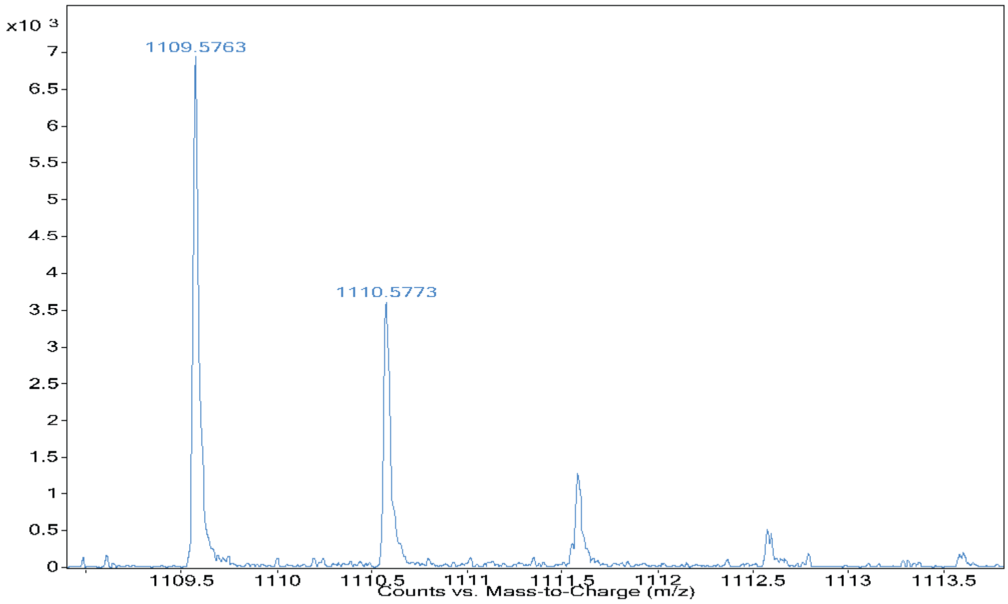

Figure 2.9. The high-resolution isotopic mass distribution of pseudomolecular ions [M+H]$^{+}$ of levorin A2.

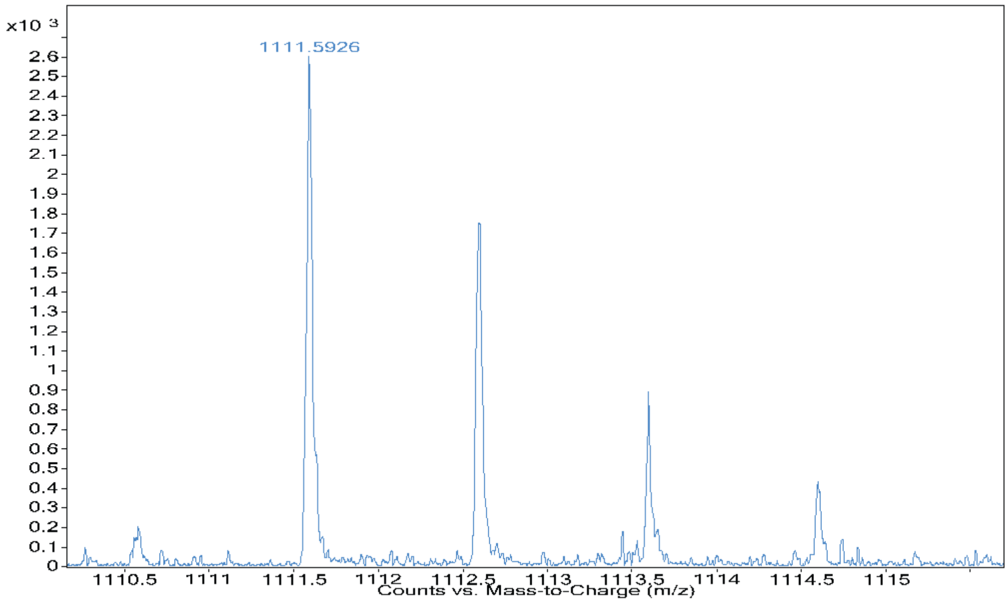

Figure 2.10. The high-resolution isotopic mass distribution of pseudomolecular ions [M+H]$^{+}$ of levorin A3.
